# Human proximal tubule epithelial cells cultured on hollow fibers: living membranes that actively transport organic cations

**DOI:** 10.1038/srep16702

**Published:** 2015-11-16

**Authors:** J. Jansen, I. E De Napoli, M. Fedecostante, C. M. S. Schophuizen, N. V. Chevtchik, M. J. Wilmer, A. H. van Asbeck, H. J. Croes, J. C. Pertijs, J. F. M. Wetzels, L. B. Hilbrands, L. P. van den Heuvel, J. G. Hoenderop, D. Stamatialis, R. Masereeuw

**Affiliations:** 1 Department of Pharmacology and Toxicology, Radboud Institute for Molecular Life Sciences, Nijmegen, The Netherlands; 2 Department of Physiology, Radboud Institute for Molecular Life Sciences, Nijmegen, The Netherlands; 3 Department of Pediatrics, Radboud university medical center, Nijmegen, The Netherlands; 4 Department of Biomaterials Science and Technology, MIRA Institute for Biomedical Technology and Technical Medicine, University of Twente, The Netherlands; 5 Department of Biochemistry, Radboud Institute for Molecular Life Sciences, Nijmegen, The Netherlands; 6 Department of Cell Biology, Radboud university medical center, Radboud Institute for Molecular Life Sciences, Nijmegen, The Netherlands.; 7 Department of Nephrology, Radboud university medical center, Nijmegen, The Netherlands.; 8 Department of Pediatric Nephrology & Growth and Regeneration, Catholic University Leuven, Leuven, Belgium.; 9 Div. Pharmacology, Department of Pharmaceutical Sciences, Utrecht University, The Netherlands.

## Abstract

The bioartificial kidney (BAK) aims at improving dialysis by developing ‘living membranes’ for cells-aided removal of uremic metabolites. Here, unique human conditionally immortalized proximal tubule epithelial cell (ciPTEC) monolayers were cultured on biofunctionalized MicroPES (polyethersulfone) hollow fiber membranes (HFM) and functionally tested using microfluidics. Tight monolayer formation was demonstrated by abundant zonula occludens-1 (ZO-1) protein expression along the tight junctions of matured ciPTEC on HFM. A clear barrier function of the monolayer was confirmed by limited diffusion of FITC-inulin. The activity of the organic cation transporter 2 (OCT2) in ciPTEC was evaluated in real-time using a perfusion system by confocal microscopy using 4-(4-(dimethylamino)styryl)-N-methylpyridinium iodide (ASP^+^) as a fluorescent substrate. Initial ASP^+^ uptake was inhibited by a cationic uremic metabolites mixture and by the histamine H2-receptor antagonist, cimetidine. In conclusion, a ‘living membrane’ of renal epithelial cells on MicroPES HFM with demonstrated active organic cation transport was successfully established as a first step in BAK engineering.

The development of biotechnological platforms to aid uremic syndrome treatment is of high interest world-wide. Current treatment strategies, such as hemo- or peritoneal dialysis, demonstrate a poor clearance of small lipid soluble and protein-bound uremic metabolites and the mortality rate in patients suffering from renal disease remains high^1^. The uremic syndrome is characterized by the retention of metabolites due to an attenuated filtration capacity of the glomerulus and a loss of function of proximal tubule epithelial cells (PTEC), which are responsible for active metabolite secretion. Elevated levels of retention solutes contribute to competitive inhibition of drug transporters and thereby alter the metabolic environment as well as drug disposition in the kidney^2^,^3^. The accumulation of uremic metabolites (i.e. uremic toxins) in the human body may contribute to further progression of renal disease and the association of chronic kidney disease with cardiovascular morbidity^4^,^5^,^6^.

A cell-aided device containing stable living membranes, which enable active transport of uremic metabolites would be a major breakthrough in the field of regenerative nephrology. Nowadays, various research groups are focussed on the development of a bioartificial kidney (BAK), containing PTEC to improve current treatment strategies^7^,^8^,^9^. In uremic animals and patients suffering from acute kidney injury (AKI) encouraging results have been obtained with respect to enhanced survival in BAK treated subjects by reduction of the inflammatory status, but further extensive research is required to obtain stable and functional cell-aided devices for chronic use^9^,^10^,^11^,^12^,^13^. A crucial step in the development of functional devices relates to advanced functionalization of the hollow fiber membranes (HFM) to stimulate monolayer formation. *In vivo*, the extracellular matrix (ECM) is key in inter- and intracellular signaling, regeneration, and support^14^,^15^. Mimicking this complex matrix to obtain well-differentiated PTEC monolayers *in vitro* is essential in BAK engineering. Successful PTEC monolayer formation was obtained using combinations of laminin, gelatin, matrigel, collagen IV and L-3,4-dihydroxydiphenylalanine (L-DOPA) ECM coatings on biomaterials^16^,^17^,^18^. However, active transport of uremic metabolites mediated by PTEC specific transporters on hollow fiber membranes to aid renal replacement therapy has, as of yet, not been demonstrated.

In addition to glomerular filtration, the renal excretion of endo- and xenobiotics is advanced by active tubular secretion, a process mediated by PTEC, which are equipped with a broad range of transporters. One of the main PTEC transporters involved in the uptake of cationic uremic metabolites and drugs, such as cimetidine, metformin and cisplatin^19^,^20^,^21^, is the basolateral organic cation transporter - 2 (OCT2; *SLC22A2*). Various cationic uremic metabolites, including guanidino compounds and polyamines, were found to interact with this transporter as well^22^. Guanidino compounds are derived from the arginine metabolism and are small, water-soluble cations and not bound to proteins^4^. However, the effective removal of guanidines is hampered in uremic conditions particularly due to their large distribution volume^23^. Guanidines are known neurotoxins^24^, but more recently the pro-inflammatory effects on the expression of leukocyte surface molecules and thus the possible interference of leukocytes with endothelial cells resulting in cardiovascular events was demonstrated as well^25^,^26^. Polyamines, derived from cellular lysine, arginine or ornithine catabolism, were identified in the late ‘70s as putative cationic uremic metabolites by Campbell *et al.*^27^. More recent work elucidated further that the polyamines cadaverine, putrescine, spermine, spermidine and their breakdown product acrolein accumulate in uremic patients and are associated with cytotoxicity, through damage to proteins, DNA and other cellular components^28^,^29^,^30^.

In our laboratory, a unique conditionally immortalized PTEC model (ciPTEC) derived from human urine was established and thoroughly characterized^31^,^32^. These cells contain the temperature sensitive vector SV40tsA58, allowing proliferation at 33 °C and differentiation in mature PTEC at 37 °C. In addition, cells were transfected with human telomerase (hTERT) limiting replicative senescence by telomere length maintenance, improving further the unlimited availability of ciPTEC. This model presents a broad range of PTEC-specific functions, including the transporters associated with uremic toxin secretion along with drug metabolism enzymes^22^,^31^,^33^. In the present study, ciPTEC monolayer function when cultured on MicroPES HFM was investigated as a crucial element of a renal assist device. To this end, permeability properties of (biofunctionalized) membranes, monolayer formation and epithelial functional characteristics were investigated. The latter included OCT2 activity assessment using a microfluidic system with real-life imaging in the presence or absence of OCT2 inhibitors as well as a cationic uremic metabolites mixture of guanidines and polyamines.

## Results

### Modified HFM maintain membrane permeability

**Figure 1:**
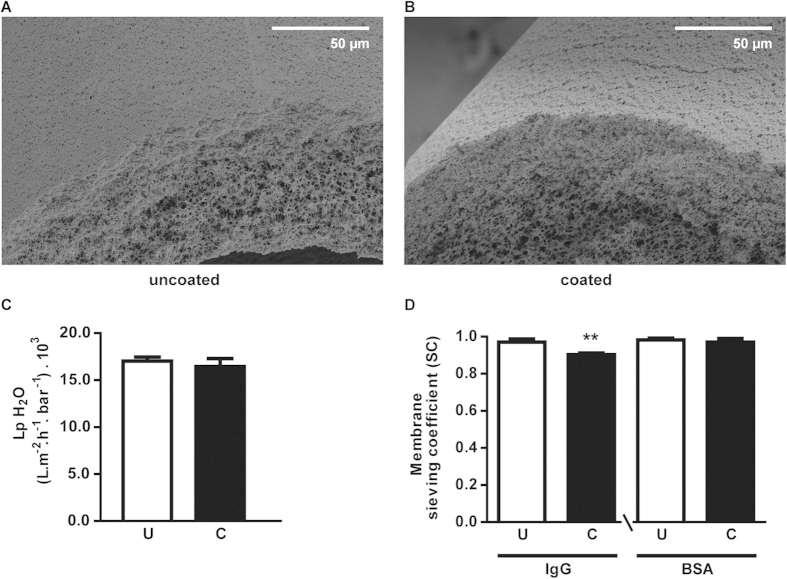
HFM topography and flux of essential compounds. (**A**,**B**) Representative SEM images of uncoated and coated MicroPES HFM are shown. No microscopic differences were observed and topography was preserved in coated HFM. (**C**) The water permeance of uncoated and coated HFM was highly similar in both conditions (U: (17.0 ± 0.3) . 10^3^ Lm^−2^ h^−1^bar^−1^) *vs.* C: (16.4 ± 0.7) . 10^3^ Lm^−2^ h^−1^bar^−1^, respectively). (**D**) The SC of IgG was slightly less in coated HFM (C: 0.90 ± 0.01, p < 0.01) compared to uncoated HFM (U: 0.97 ± 0.02), whereas BSA passed almost freely through the membrane as demonstrated by the SC close to 1 for coated (C: 0.97 ± 0.02) and uncoated HFM (U: 0.98 ± 0.01). Data are shown as mean ± S.E.M. of three independent experiments performed in duplicate ** p < 0.01 using an unpaired *t* test.

The surface of MicroPES HFM was functionalized via a double coating to enable tight homogeneous cell monolayer formation. The coating was successfully developed in 2D by our group using Transwell® membrane supports^18^. Here, this was optimized further in 3D. The membrane surface and cross sections of uncoated *and* coated HFM in the absence of cells were investigated using SEM (Fig. 1A,B). In agreement with our previous study^18^, the coatings applied were thin and did not differ between fibers, consistent with their transport properties. The coated HFM should facilitate transepithelial transport of protein-bound uremic toxins, with retained solute permeability. As shown in Fig. 1C, the H_2_O permeability was preserved in double coated HFM ((16.4 ± 0.7) .10^3^ L m^−2^ h^−1^bar^−1^) compared to uncoated HFM ((17.0 ± 0.3) . 10^3^ L m^−2^ h^−1^bar^−1^). In addition, both IgG and BSA passed almost freely through the membrane as demonstrated by the sieving coefficient (SC) close to 1 for both coated (0.90 ± 0.01 and 0.97 ± 0.02, respectively; p < 0.01) and uncoated HFM (0.97 ± 0.02 and 0.98 ± 0.01, respectively) (Fig. 1D). Obviously, the final composition of coated HFM with a tight cell monolayer should form a true barrier and prevent the loss of essential components such as IgG and albumin into the dialysate.

### Extracellular matrix, cellular adhesion and paracellular permeability

**Figure 2:**
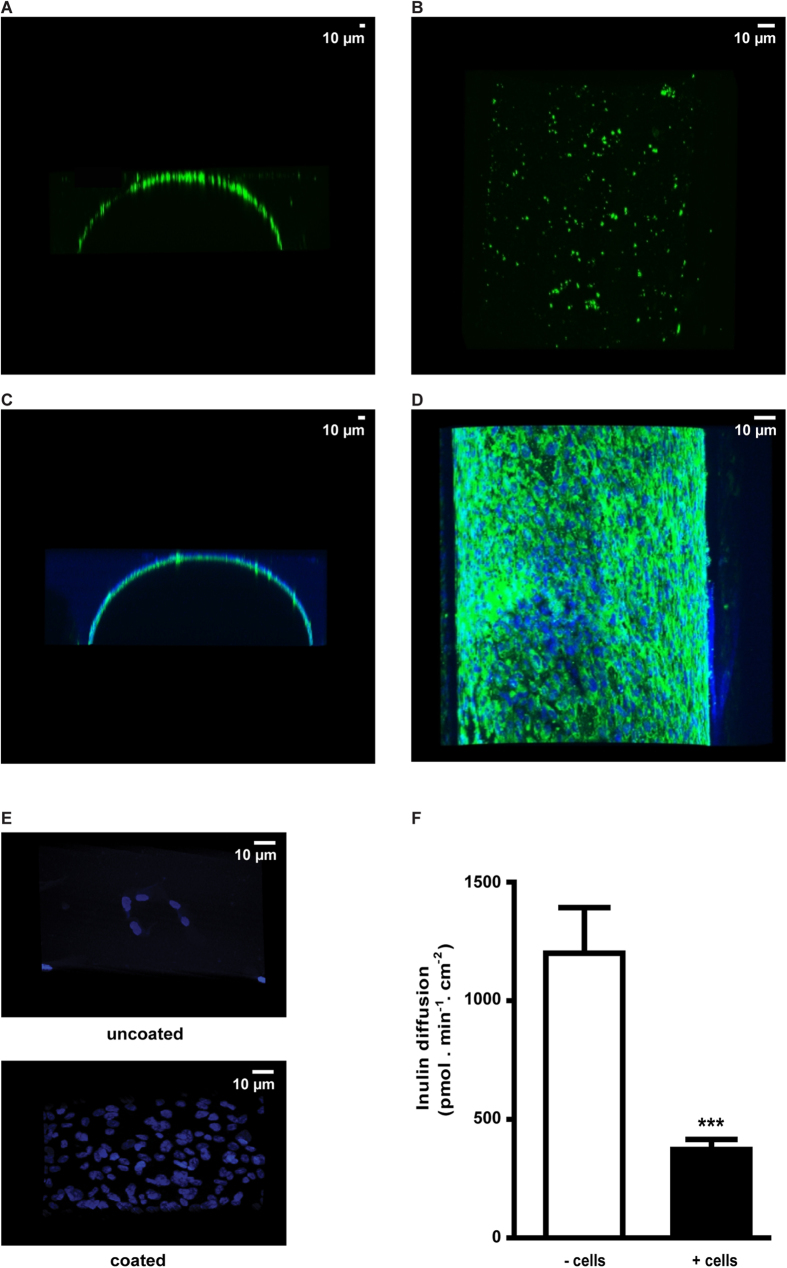
Extracellular matrix, cellular adhesion and paracellular permeability. Representative confocal images of collagen IV (green) in y-z (**A**,**C**) and x-y (**B**,**D**) planes are shown. (**A**,**B**) A heterogeneous collagen IV ECM pattern was detected on coated HFM without cells. (**C**,**D**) Nuclei were stained using DAPI (blue) in matured ciPTEC cultured on coated HFM. A homogenous and abundant distribution of collagen IV (green) was detected in the presence of matured ciPTEC. (**E**) Representative images of adhered cells (matured ciPTEC) are shown by nuclei staining (dapi; blue). A limited number of cells were present when cultured on uncoated HFM whereas numerous cells adhered to the coated HFM surface. (**F**) The transepithelial barrier function was examined in the presence or absence of matured ciPTEC by inulin-FITC perfusion and the flux across the HFM was quantified. Data are shown as mean ± S.E.M. of three independent experiments performed in triplicate or duplicate. *** p < 0.001 using an unpaired *t* test.

Collagen IV is a non-fibrillar collagen and is known to assist in cell differentiation towards renal lineages from various stem cell populations^34^. The presence of a collagen IV matrix after the coating procedure was confirmed, as shown in Fig. 2A,B, though a heterogeneous pattern was observed. Interestingly, in the presence of matured ciPTEC, a nice homogeneous collagen IV matrix was visible (Fig. 2C,D). Moreover, cell adhesion clearly improved upon surface modification, as shown in Fig. 2E, suggesting biofunctionalization. Hardly any nuclei (*i.e.* cells) were detected when cultured on uncoated HFM, whereas a uniform distribution of nuclei was observed after double coating. Furthermore, the transepithelial barrier function was quantified by inulin-FITC diffusion after perfusion of HFM in the presence or absence of matured ciPTEC. As shown in Fig. 2F, matured ciPTEC formed a true barrier compared to coated HFM in the absence of cells (373 ± 42 pmol min^−1^ cm^−2^; 1200 ± 193 pmol min^−1^ cm^−2^, respectively, p < 0.001). In addition, cell adhesion of ciPTEC to PES HFM was found to be related to the curvature of the membrane. Similar double coating procedures applied to PES HCO 1100 membranes with an inner diameter of 215 μm resulted in poor ciPTEC adherence (Figure S2), in contrast to double coated MicroPES HFM with an inner diameter of 300 μm (Fig. 2).

### Intact cellular organelles and numerous microvilli

**Figure 3:**
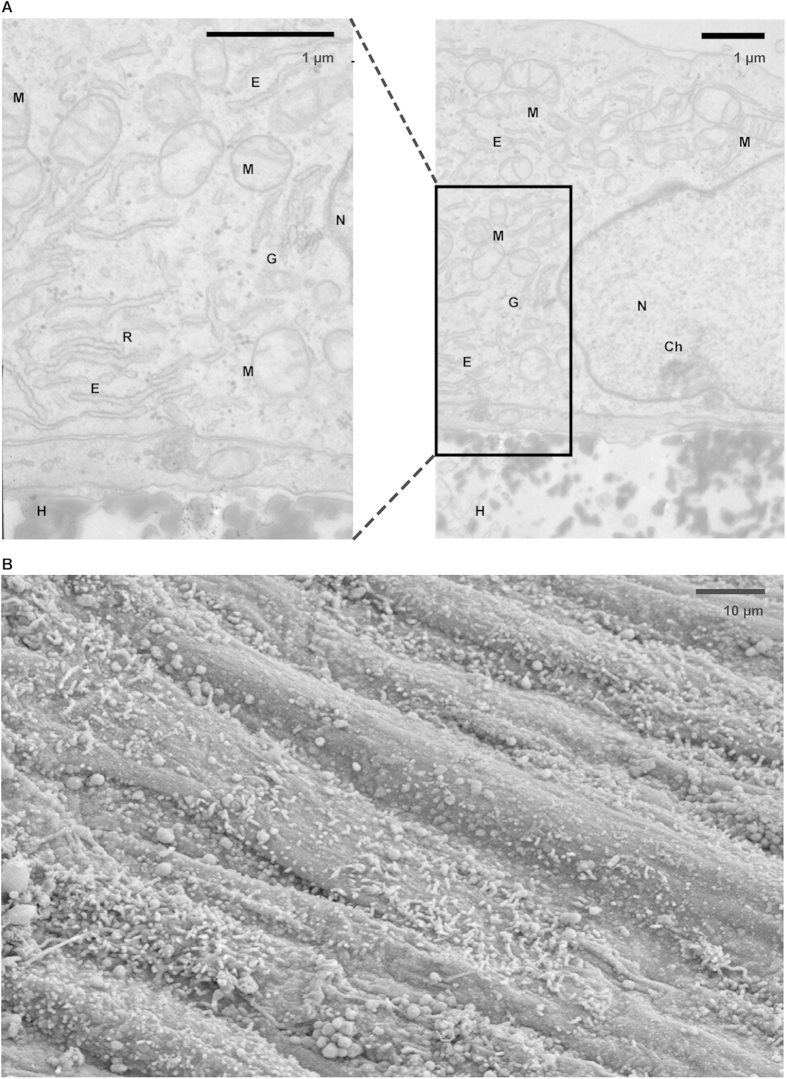
Cellular organelles and surface characteristics. (**A**) The morphology of organelles in matured ciPTEC cultured on HFM (H) was investigated by transmission electron microscopy and intact cells were detected. Next to numerous well-developed mitochondria, the nucleus (**N**) containing the chromatin (Ch), the endoplasmatic reticulum (**E**), ribosomes (**R**) and the Golgi apparatus could be nicely detected. (**B**) The surface of matured ciPTEC was visualized using scanning electron microscopy. At the apical membrane microvilli were observed, as expected for proximal tubule epithelial cells, though a heterogeneous distribution was detected.

Matured ciPTEC cultured on HFM retained intact cellular organelle morphology as observed by TEM (Fig. 3A). The presence of numerous mitochondria indicated that the cell monolayer consists of viable and metabolically active PTEC^35^. Moreover, other essential cellular components, including the Golgi apparatus, endoplasmatic reticulum, ribosomes, the nucleus and its chromatin, could be determined. The brush border membrane of ciPTEC is reasonably well-developed, containing many microvilli to enlarge the apical surface and to stimulate intensive reabsorption, as observed with SEM (Fig. 3B).

### Homogenous cell monolayers represent ZO-1 and OCT2 expression

**Figure 4:**
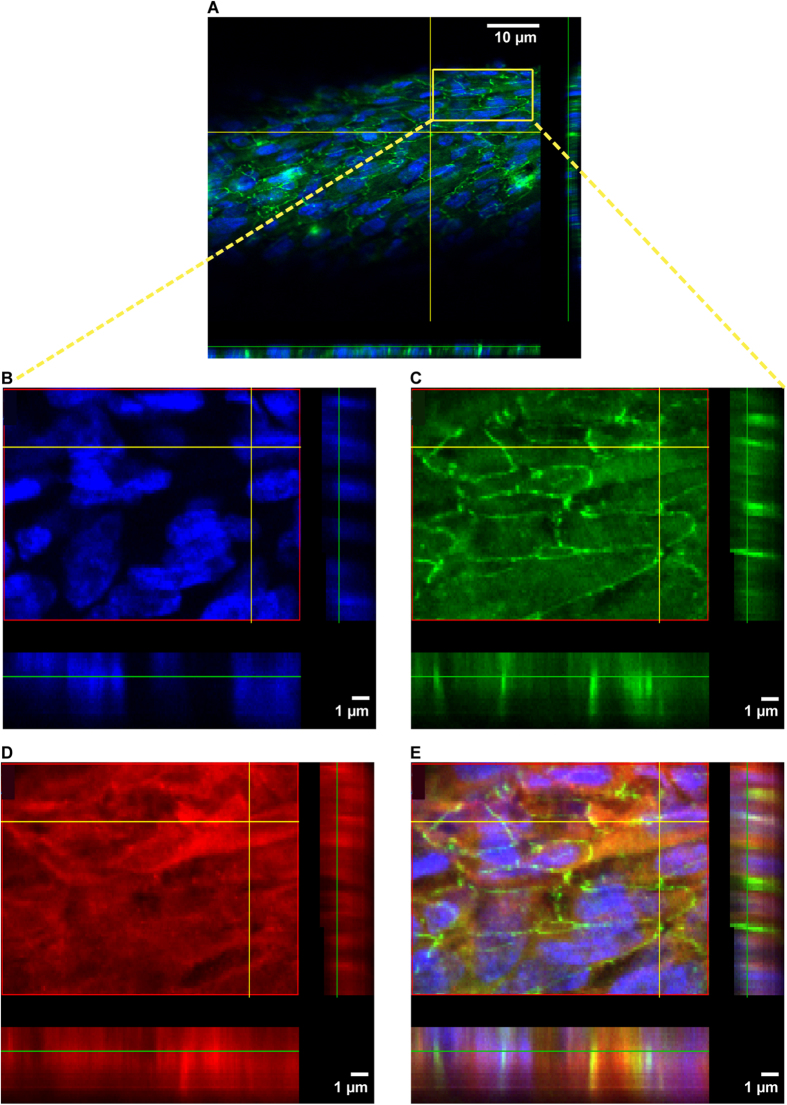
ZO-1 and OCT2 protein expression. Representative confocal images of ZO-1 (green), OCT-2 (red) and nuclei (blue) in matured ciPTEC cultured on HFM are shown in x-y and y-z planes. (**A**–**C**,**E**) The ZO-1 expression was abundantly present along the cell boundaries within a tight and homogenous cell monolayer. (**D**,**E**) The endogenous OCT-2 protein expression in ciPTEC was observed heterogeneously in the membranes, moreover, some signal was detected in the cytoplasm.

The presence of the tight junction protein ZO-1 in cell monolayers emphasizes cell polarity and monolayer tightness. In addition, tight junction proteins contribute to fluid and ion homeostasis mediated by paracellular transport^36^. A tight homogeneous cell monolayer was observed for ciPTEC on HFM with ZO-1 abundantly expressed along the cell boundaries as shown in representative z-scans (Fig. 4A,C). Moreover, the endogenous expression of the influx transporter OCT2 was demonstrated to be expressed basolaterally (Fig. 4D), although some cytoplasmic staining was visible as well, suggesting ongoing posttranslational OCT2 modifications as previously suggested^37^. The OCT2 antibody was validated in paraffin embedded human kidney tissue using immunohistochemistry and positive staining at the basolateral membrane of PTEC was observed (data not shown).

### ciPTEC on HFM show functional OCT2 transport

**Figure 5:**
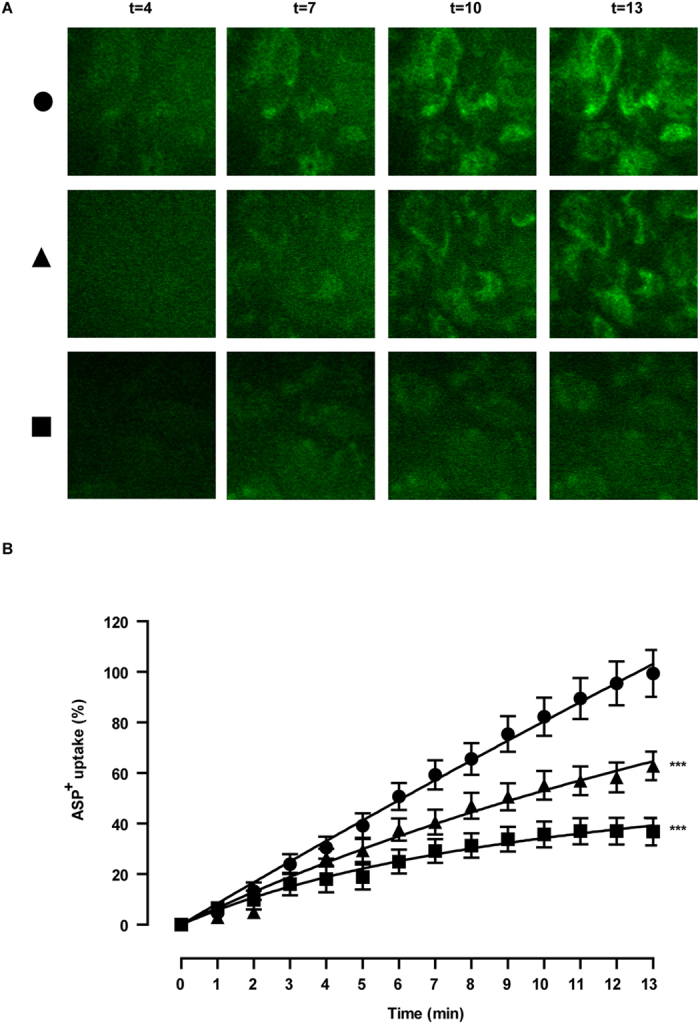
Functional organic cation transporter - 2 transport. (**A**) Representative real-time images and (**B**) semi-quantification of ASP^+^ uptake (10 μM, green) are shown, a model substrate for OCT-2, in the absence (circle) or presence of specific inhibitors (triangle: cationic uremic toxin mix (UTmix), square: cimetidine (100 μM)) in matured ciPTEC cultured on HFM. Data were normalized against ASP^+^ uptake in the absence of inhibitors and were fitted according to One-phase exponential association using nonlinear regression analysis. (a - circle) After 4 min, intracellular ASP^+^ signal could be visualized and further increased until 13 min. (a - triangle) In the presence of UTmix the ASP^+^ uptake was less pronounced during the complete uptake experiment compared to perfusion with only ASP^+^. (a - square) The ASP^+^ uptake was clearly attenuated in the presence of cimetidine and hardly any further increase in fluorescent ASP^+^ uptake was detected after 7 min. (**B**) Semi-quantification of ASP^+^ uptake data in the absence (10 μM, circle) or presence of specific inhibitors (triangle: cationic uremic toxin mixture (UTmix, (10×)), square: cimetidine (100 μM) in matured ciPTEC cultured on HFM are shown. A clear fluorescent intracellular signal in ciPTEC indicated an active OCT-2 mediated basal ASP^+^ uptake started at 1 min. Initial uptake was significantly inhibited by the UT mixture and cimetidine. Data are shown as mean ± S.E.M. of three independent experiments performed in duplicate. Slopes extracted from min 1–8 of the ASP^+^ uptake in different conditions were compared and applied for statistical analysis. ***p < 0.001 using two-way ANOVA analysis followed by Bonferroni post-tests.

The activity of basolaterally expressed OCT2 in matured ciPTEC cultured on coated HFM was determined by real-time fluorescent ASP^+^ uptake measurements, and shown in Fig. 5. The experiments were performed in the presence or absence of a cationic toxin mix or cimetidine, which are known inhibitors of OCT2-mediated ASP^+^ transport^22^. In Fig. 5A, representative real-time uptake images of the various conditions are shown for the time frame of 4–13 min. In the absence of inhibitors, a clear intracellular fluorescent ASP^+^ signal was visible after 4 min of perfusion, which increased up to 13 min. In the presence of the cationic uremic toxin mixture of guanidine and polyamine compounds, the basal uptake of ASP^+^ was still detectable and augmented over time, though the uptake was less pronounced compared to fiber perfusion in absence of the mixture. In the presence of cimetidine, the ASP^+^ uptake was even further attenuated. Semi-quantification of these data (Fig. 5B) confirmed the observed differences in ASP^+^ uptake per condition. The slopes from the linear part (min 1–8) of ASP^+^ uptake curves in the presence of the cationic toxin mix or cimetidine were significantly altered compared to control uptake (25 ± 16%, p < 0.001 and 62 ± 12%, p < 0.001, respectively).

## Discussion

In this study, ciPTEC were successfully cultured on optimally coated HFM with maintained functionality. The coating and membrane curvature showed to be crucial for the formation of a homogenous cell monolayer with a clear epithelial barrier function, without affecting transport properties of H_2_O or albumin. Epithelial cell polarity and organelle morphology were retained when cultured on HFM. In addition, the epithelial and polarized characteristics of ciPTEC were demonstrated further by the expression of ZO-1 and the basolaterally expressed OCT2. Transport activity was confirmed by specific uptake in ciPTEC monolayers, demonstrating the establishment of a living membrane suitable for BAK development.

The MicroPES membranes used in this study are hydrophilized fibers used predominantly for plasma separation and have rather large pores allowing the transport of proteins including BSA and IgG. The membranes are designed for having low interaction with blood, plasma and proteins. To apply a uniform cell monolayer, the outer membrane of HFM had to be biofunctionalized by ECM components. The native ECM is a dynamic network consisting of various types of collagen, glycosaminoglycans, laminin and fibronectin connected to integrins present in the plasma membrane. The ECM is a key factor in cell adhesion, differentiation and regeneration, as well as in intercellular signalling^14^,^15^. Oo *et al.* demonstrated a successful coating on PES membranes consisting of the combination of L-DOPA and collagen IV to culture primary human renal cells^38^. Upon polymerization, when exposed to light and oxygen, the L-DOPA layer becomes negatively charged and will attract the positively charged collagen IV that is applied subsequently. In our laboratory, this coating was first applied on flat PES membranes and optimal conditions for ciPTEC adhesion and differentiation were established^18^. Maintained membrane permeability, as well as homogenous cell monolayers and creatinine transport were demonstrated in ciPTEC on double coated PES flat membranes. Based on these encouraging results, the coating was adapted for HFM biofunctionalization. Both L-DOPA and collagen IV coating times were extended to hours instead of minutes to stimulate cell adhesion and tight cell monolayer formation. Next to an optimized ECM, the membrane curvature is an important parameter for maintaining cellular functions^39^. The culture of ciPTEC on MicroPES HFM with a slightly lower membrane curvature compared to the HCO 1100 HFM had a positive effect on cell adhesion, most likely related to a more optimal organization of the cytoskeleton and its membrane composition. The almost unrestricted permeability of H_2_O, IgG and albumin in coated MicroPES HFM indicated the need for a tight cell monolayer with a true barrier function to avoid significant blood components loss from the host. Hence, the free permeability of albumin across coated HFM is essential, as a large number of uremic toxins are protein-bound and need to be delivered in close proximity to the cells in order to be eliminated. Possible immunogenic effects due to contact between the host blood components and cell monolayers will be studied thoroughly in future research.

The morphological characteristics of tight cell monolayers on HFM were examined, as well as the epithelial barrier functions. *In vivo*, the proximal tubule epithelium is known as a leaky epithelium and paracellular transport is mediated along the tight junctions and lateral inter-cellular spaces^35^. The reabsorption of glucose, amino acids, H_2_O and electrolytes is partly facilitated by paracellular transport and occurs in a passive manner. The abundant expression of the tight junction protein ZO-1 in the monolayers emphasized the epithelial character of the ciPTEC when cultured on HFM. Moreover, the epithelial barrier function was confirmed by demonstrating limited inulin-FITC diffusion. Since technical aspects of fiber handling when placed in the perfusion chamber will affect the tightness of the cell layer, the barrier restriction measured, as shown here, is most likely an underestimation. To prevent monolayer disturbance caused by technical aspects in future research, cell culture on HFM could be performed in disposable perfusion chambers. In this way, the cells on HFM will mature while already present in the system and will be ready-to-use for any perfusion experiment. Vital PTEC possess a high number of mitochondria to facilitate active processes (e.g. elimination of waste products) and a well-developed brush border membrane containing microvilli to enlarge the apical surface^35^. The morphology of ciPTEC reflected the presence of intact organelles and a substantial number of mitochondria. The microvilli at the apical surface were visible when ciPTEC were cultured on HFM, but showed a heterogeneous pattern. Further optimization of culture conditions in disposable perfusion chambers, including medium flow across the apical membrane, could improve the uniformity of the ciPTEC brush border membrane^40^.

The application of a cell-aided device, such as the BAK, for the removal of cationic metabolites can improve current renal replacement therapies. In this study, active OCT2 transport was demonstrated in living membranes of human PTEC when cultured on HFM for BAK purposes. Uptake via the OCT2 transporter is the first step in the renal secretion of various cationic endogenous metabolites or other xenobiotics, including drugs^41^. Upon transport failure, accumulation of cationic metabolites, such as guanidines occurs. This is associated with cardiovascular implications, induced by, amongst others, activated leukocytes and microinflammation^25^,^26^. Here, OCT2-mediated ASP^+^ uptake was inhibited in the presence of a polyamine and guanidino cationic uremic toxin mixture, as well as in the presence of cimetidine, a well-known and extensively studied renal OCT substrate and inhibitor^42^,^43^. These findings indicate functional OCT2 transport in ciPTEC on HFM, in concordance with recently described observations by Schophuizen *et al.*, where the cationic toxin mixture competed with OCT2-mediated ASP^+^ uptake in a ciPTEC cell suspension^22^. In addition, Kimura and Winter *et al.* demonstrated interference of guanidino compounds and putrescine, a polyamine, with OCT2 using human embryonic kidney cell line (HEK293) overexpressing the transporter^44^,^45^. The application of functional ciPTEC cultured on HFM opens a promising avenue in the clearance of cationic metabolites in patients suffering from renal disorders. Future research is required to quantify transepithelial transport of uremic metabolites in up-scaled multifiber bioreactors. As (cationic) uremic toxins compete for the same system, their accumulation may also hamper efficient removal. Therefore, in-depth kinetic studies are needed to investigate the clearance capacity of our bioartificial tubule over time, and to predict their performance *in vivo*. In addition, other aspects, such as the balance between the number of ciPTEC and the duration per dialysis cycle required for efficient removal of waste products in human patients, have to be investigated.

The development of a functional and stable BAK device is a complex interplay between advanced biomaterials and renal epithelial cells. Beneficial effects were demonstrated in uremic animals and enhanced survival of patients suffering from multi-organ failure and AKI in a phase II clinical trial. However, a phase IIb clinical trial failed predominantly due to hampered cell performance^9^,^10^,^11^,^12^,^13^,^46^. In the late ‘90s, Humes *et al.* cultured porcine proximal tubule epithelial cells (LLC-PK1) in a multi-fiber bioreactor and demonstrated metabolically active cells, capable of ammoniagenesis, glutathione metabolism and 1,25-dihydroxyvitamin D_3_ synthesis^47^. However, next to its non-human origin, porcine cells have limited transport functionality (e.g. lack of essential transporters^48^,^49^) and are not preferred for use in a BAK device. Only a few human-derived cell models are available with endogenous transporter expression and functional capacity. A well-distributed cell line is HK2, which was developed by recombinant retroviral transduction (humanpapillomavirus 16 E6/E7 genes) of human primary renal cortex cells^50^, though a lack of functional transporters emphasizes the limitations of this model for studying renal transport in pharmacological and physiological studies^51^. The thoroughly characterized cell model ciPTEC, as used in this study, was isolated from human urine and immortalized using retroviral hTERT and SV40 large T antigen transduction^32^. The cell model displays diverse functional endogenous in- and efflux transporters relevant in the secretion of endo- and xenobiotics^31^,^32^. In addition, UDP-glucuronosyltransferases (UGT) and mitochondrial succinate dehydrogenase enzyme activity was confirmed and altered in uremic conditions that might affect drug disposition during CKD^33^. Similar to HK-2, no organic anion transporter (OAT) expression could be detected in ciPTEC, a known phenomenon of immortal renal cells in culture and a drawback of renal cell line applications^52^. Nevertheless, the stable functionality of other relevant transporters and metabolic enzymes in ciPTEC upon prolonged culturing illustrate that these cells may prove a valuable tool in regenerative nephrology. Furthermore, this model may serve as a predictive model for drug toxicity screening in drug development processes.

To treat patients suffering from CKD or end-stage renal disease (ESRD), an efficient removal of protein-bound uremic toxins by a BAK is a prerequisite to diminish systemic disease progression^53^. The active transport of cationic uremic toxins mediated by ciPTEC, as shown here, is an important contribution for future BAK engineering. However, future research is required to study the maintenance of functional cationic and anionic toxin transport in an up-scaled BAK system, as well as multi-factorial safety aspects, as described by the European medicines agency in the European guidelines for advanced therapy medicinal products enabling the applications of GMO in medicinal products^54^.

In conclusion, a successful cell monolayer formation was established on biofunctionalized MicroPES HFM and matured ciPTEC represented clear epithelial characteristics with barrier and active transporter function. To the best of our knowledge, for the first time active organic cationic transport in PTEC monolayers on HFM was demonstrated. The combination of a unique human cell model with biofunctionalized MicroPES HFM is a valuable tool in regenerative nephrology and further development may contribute in future BAK engineering.

## Methods

Brief methods are given. For details, the reader is referred to Supplementary materials.

### Chemicals and cell culture materials

Chemicals were purchased from Sigma-Aldrich (Zwijndrecht, The Netherlands) unless stated otherwise. MicroPES type TF10 hollow fiber capillary membranes (wall thickness 100 μm, inner diameter 300 μm, max pore size 0.5 μm) were obtained from Membrana GmbH (Wuppertal, Germany). Cell culture plates were purchased from Greiner Bio-One (Monroe, NC).

### Hollow fiber sterilization and double coating

MicroPES (polyethersulfone) hollow fiber membranes were sterilized using 70% (v/v) EtOH incubation for 30 min. The primary coating component L-DOPA (L-3,4-dihydroxyphenylalanine, 2 mg.ml^−1^) was dissolved in 10 mM Tris buffer (pH 8.5), as described previously by Ni *et al.*^16^, at 37 °C for 45 min. Next, the solution was filter sterilized prior to incubation of the horizontally placed fibers for 5 hours at 37 °C. The L-DOPA coated fibers were exposed to the second coating component human collagen IV (C6745-1 ml, 25 μg.ml^−1^) for 1 hour at 37 °C. The collagen IV solution was aspirated afterwards and the fibers were washed thoroughly in HBSS buffer prior to cell seeding.

### Scanning electron microscopy of cell-free hollow fiber membranes

Membrane topography of uncoated and coated HFM was determined using scanning electron microscopy (SEM) analysis (Philips XL-30 ESEM, Amsterdam, the Netherlands). In short, HFM were prepared using liquid nitrogen fracture to enable cross section analysis. Samples were incubated at 37 °C overnight and gold sputtered before examination by SEM (3 kV and 5 kV).

### Transport properties of cell-free hollow fibers membranes

The transport of pure water (Merck MilliPore, Billerica, MA), bovine serum albumin (BSA) and immunoglobulin G (IgG) solutions through the uncoated and coated cell-free HFMs were measured at various transmembrane pressures using a KrosFlo Research IIi Tangential Flow Filtration system (Spectrum laboratories, Wurzburg, Germany). The membrane permeability of H_2_O was plotted as the slope of the flux versus the transmembrane pressure. The membrane sieving coefficient (SC) was calculated by dividing the concentration of the BSA and IgG in the permeate by the concentration of the feed protein solution.

### Culture of ciPTEC on double coated hollow fiber membranes

Proliferating ciPTEC^32^ were seeded on double-coated fibers (length 2 cm) using 1.5 × 10^6^ cells per 1.5 ml. The cell suspension was added to an eppendorf tube containing the fiber and incubated at 33 °C, 5% (v/v) CO_2_, for 4 hours. Every hour the tube was turned 90° in order to stimulate cell adhesion to the whole surface. Next, fibers with adhered cells were removed from the suspension and transferred to a 6 well plate with PTEC culture medium and were cultured as previously described by Jansen *et al.*^31^.

### Immunocytochemistry

The expression of OCT2, zonula occludens-1 (ZO-1) and the ECM component collagen IV in ciPTEC monolayers on hollow fibers were investigated using the immunocytochemistry methodology as previously described by Jansen *et al.*^31^. The expression and localization of the proteins of interest were examined using the Olympus FV1000 Confocal Laser Scanning Microscope (Olympus, Tokyo, Japan) and images were captured using the Olympus software FV10-ASW version 1.7.

### Transmission- and scanning electron microscopy

The morphology of ciPTEC and its organelles was investigated using transmission electron microscopy (TEM) and SEM. In short for TEM, cells were fixed and dehydrated using series of chemical solutions and finally embedded in Epon and polymerized at 60 °C. After ultrathin sectioning and contrasting with uranyl acetate and lead citrate, sections were analyzed using a transmission electron microscope JEOL JEM 1010 (Jeol, Akishima Tokyo, Japan). For SEM purposes, cells were fixed and dehydrated using various chemical solutions and finally critical point dried. Samples were gold sputtered prior to SEM analysis (JEOL JSM-6340F, Tokyo, Japan).

### Transepithelial barrier function

Paracellular permeability was determined in the living membrane by endpoint quantification of inulin-fluorescein isothiocyanate (FITC) (0.1 mg.ml^−1^ in Krebs-Henseleit buffer) supplemented with HEPES (10 mM; KHH buffer)) diffusion when perfused (6 ml.h^−1^, in close agreement with previously applied flow rates^8^) using a custom-made microfluidic system (supplemental figure S1) connected to a syringe pump (Terumo STC-521, Terumo Europe N.V., Leuven, Belgium) for 13 min at 37 °C. As a control, the inulin-FITC diffusion in coated HFM in the absence of cells was investigated in similar conditions. Fluorescence was detected by measuring samples and a standard range (0.0005–0.1 mg/ml, including a final blank sample (KHH buffer) 100 μl) at excitation wavelength 485 nm and emission wavelength 535 nm, using a Victor^TM^ X3 multilabel platereader (Perkin-Elmer, Groningen, The Netherlands). Blank data were subtracted from all arbitrary fluorescence units measured and the corrected AFU data of the standard range were used to calculate the inulin-FITC concentration of the samples (mg/ml). Next, the flux (*J*) was calculated according to:


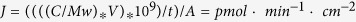


where C is the calculated concentration (mg/ml), Mw the average inulin-FITC molar mass in mg/mmol (4500 mg/mmol), V the volume present in the apical compartment of the perfusion chamber (0.3 ml), t the perfusion time (13 min) and A the fiber surface (0.13 cm^2^).

### Functional organic cation transport

The activity of OCT2 in ciPTEC cultured on HFM was examined real-time by perfusing the hollow fiber basolaterally (inner HFM, (6 ml.h^−1^,^8^)) with the fluorescent OCT2 substrate 4-(4-(dimethylamino)styryl)-N-methylpyridinium iodide (ASP^+^, 10 μM) in KHH buffer, when assembled in a microfluidic system. The assay was performed in the presence or absence of a cationic uremic toxin mix (UTmix; 10 times uremic plasma concentrations reported in literature - spermidine 6.7 μM, spermine 0.9 μM, cadaverine 2.1 μM, putrescine 8.8 μM, acrolein 14.2 μM, guanidine 21.8 μM and methylguanidine 76.6 μM)^22^ or cimetidine (100 μM), for 13 min at 37 °C, 5% (v/v) CO_2_. Imaging was performed using the Zeiss LSM510 META microscope (Zeiss, Oberkochen, Germany). Semi-quantification of real-time data was performed using Image J software (version 1.40 g). Of each fiber in time spanning 1–13 min (800 scans), four cells present in the focal plane were used to extract mean ASP^+^ pixel intensities and background was subtracted using pixel intensities form ASP^+^ perfused non-cell double-coated fibers. Next, data were normalized according to:


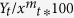


where Y_*t*_ is the pixel intentsity at time point *t* of a condition (ASP^+^ only, ASP^+^ + UTmix or ASP^+^ + cimetidine) divided by X^*m*^_*t*_ , which is the average pixel intensity (*m*) of only ASP^+^ condition at that similar time point (*t)*. This value was multiplied by 100 and the obtained relative data were fitted according to One-phase exponential association using nonlinear regression analysis. To compare ASP^+^ uptake in different conditions, slopes were extracted from min 1–8 and compared. In detail, of each fiber from min 1–8 (428 scans), four cells present in the focal plane were used to extract mean ASP^+^ pixel intensities and were background corrected. Average pixel intensities from these four cells per fiber were plotted and six slopes derived from six fibers per condition were calculated using linear regression analysis and compared to ASP^+^ uptake using two-way ANOVA analysis followed by Bonferroni post-test.

### Data analysis

All data are expressed as mean ± S.E.M of multiple replicates. Nonlinear curve fitting and statistical analysis of functional organic cation data were performed with GraphPad Prism version 5.02 (La Jolla, CA). A two-way ANOVA analysis followed by Bonferroni post-test or, when appropriate, an unpaired *t* test was applied. A p-value of <0.05 was considered significant.

## Additional Information

**How to cite this article**: Jansen, J. *et al.* Human proximal tubule epithelial cells cultured on hollow fibers: living membranes that actively transport organic cations. *Sci. Rep.* **5**, 16702; doi: 10.1038/srep16702 (2015).

## Supplementary Material

Supplementary Information
