# Supplementary material for: Human proximal tubule epithelial cells cultured on hollow fibers: living membranes that actively transport organic cations
Source: Sci Rep. 2015 Nov 16;5:16702. doi: 10.1038/srep16702 (PMC4644946; doi:10.1038/srep16702)
Supplement: Supplementary Information [file srep16702-s1.pdf]

Human proximal tubule epithelial cells cultured on hollow fibers: living membranes that actively transport organic cations

Supplementary material

Jansen J[123], De Napoli IE[4], Fedecostante M[123], Schophuizen CMS[123], Chevtchik NV[4], Wilmer MJ[1], van Asbeck AH[5], Croes HJ[6], Pertijs JC[1], Wetzels JFM[7], Hilbrands LB[7], van den Heuvel LP[3,8], Hoenderop JG[2], Stamatialis D[4], Masereeuw R[19†]

Department of [1]Pharmacology and Toxicology, [2]Physiology, [5]Biochemistry and [6]Cell Biology, Radboud university medical center, Radboud Institute for Molecular Life Sciences, Nijmegen, The Netherlands.

Department of [3]Pediatrics and [7]Nephrology, Radboud university medical center, Nijmegen, The Netherlands.

Department of [4]Biomaterials Science and Technology, MIRA Institute for Biomedical Technology and Technical Medicine, University of Twente, The Netherlands

Department of [8]Pediatric Nephrology & Growth and Regeneration, Catholic University Leuven, Leuven, Belgium.

Div. [9]Pharmacology, Department of Pharmaceutical Sciences, Utrecht University.

[†] Corresponding author:

Rosalinde Masereeuw, Ph.D.

Utrecht University

Div. Pharmacology, Dept. of Pharmaceutical Sciences

Universiteitsweg 99

3584 CG Utrecht, The Netherlands

r.masereeuw@uu.nl

Phone: +31-30-253-3529

Fax: +31-30-253-7900

Methods

Hollow fiber sterilization and double coating

MicroPES (polyethersulfone) hollow fiber membranes were sterilized using 70% (v/v) EtOH incubation for 30 min. Subsequently, membranes were washed thoroughly in Hank's balanced salt solution (HBSS) buffer containing $CaCl_2$ (1.26 mM), MgCl (0.49 mM), $MgSO_4$ (0.41 mM), KCl (5.33 mM), KH_2PO_4 (0.44 mM), $NaHCO_3$ (4.17 mM), NaCl (137.93 mM), Na_2HPO_4 (0.34 mM) and D-Glucose (5.56 mM). The primary coating component L-DOPA (L-3,4-dihydroxyphenylalanine, 2 $mg.ml^{-1}$) was dissolved in 10 mM Tris buffer (pH 8.5), as described previously by Ni *et al.* [16]. To dissolve the coating, the solution was incubated at 37°C for 45 min. Next, the solution was filter sterilized prior to incubation of the horizontally placed fibers for 5 hours at 37°C. Every hour the fiber was turned 90°. Next, the L-DOPA solution was aspirated and the fibers were washed once with HBSS buffer. The L-DOPA coated fibers were exposed to the second coating component human collagen IV (C6745-1ml, 25 $\mu g.ml^{-1}$) for 1 hour at 37°C. After 30 min, the tube was turned 180° and incubated for the remaining time. The collagen IV solution was aspirated afterwards and the fibers were washed thoroughly in HBSS buffer prior to cell seeding.

Transport properties of cell-free hollow fibers membranes

The transport of pure water (Merck MilliPore, Billerica, MA), bovine serum albumin (BSA) and immunoglobulin G (IgG) solutions through the uncoated and coated cell-free HFMs were measured at various transmembrane pressures using a KrosFlo Research Iii Tangential Flow Filtration system (Spectrum laboratories, Wurzburg, Germany). Prior to the experiment, HFMs were incubated at 37°C in phosphate buffered saline, (PBS, pH 7.4; Life technologies, Breda, The Netherlands) for 1 hour. Next, the HFMs were assembled in one-membrane module using bi-component polyurethane potting glue (Intercol BV, Ede, The Netherlands), push-in fittings (Festo, Brussels, Belgium) and polyethylene tubing (Festo, Brussels, Belgium). The BSA and IgG were dissolved in PBS at concentrations of 1 $mg.ml^{-1}$ and 0.02 $mg.ml^{-1}$, respectively (feed solutions). The pressure (0.10, 0.15 and 0.20 bar) was applied from the luminal side towards the abluminal side of the HFM. As described previously by Schophuizen *et al.* [18], the flux through the HFM at each pressure was determined by collecting the mass of the permeated liquid over time correlated to the HFM surface area. BSA and IgG samples were collected at each pressure after 15 min and concentrations were determined by spectrophotometric analysis (Cary 300 UV-Vis system, Agilent Technologies, Amstelveen, The Netherlands) at 280 nm and 276 nm wavelengths, respectively.

Culture of ciPTEC on double coated hollow fiber membranes

Proliferating ciPTEC [32] were seeded on double-coated fibers (length 2cm) using 1.5 x 10^6 cells per 1.5 ml. The cell suspension was added to an eppendorf tube containing the fiber and incubated at 33°C, 5% (v/v) CO_2, for 4 hours. Every hour the tube was turned 90° in order to stimulate cell adhesion to the whole surface. Next, fibers

with adhered cells were removed from the suspension and transferred to a 6 well plate with PTEC culture medium: phenol-red free DMEM-HAM's F12 medium (catalogue number 11039, Lonza, Basel, Switzerland) containing 10% (v/v) FCS (Greiner Bio-One, Monroe, NC), 5 µg/ml insulin, 5 µg/ml transferrin, 5 ng/ml selenium, 36 ng/ml hydrocortisone, 10 ng/ml EGF and 40 pg/ml tri-iodothyronine. The incubation period was prolonged at 33°C, 5% (v/v) CO_2, for additional 48 hours. Subsequently, the proliferating cells on fibers were transferred to 37°C, 5% (v/v) CO_2 to mature for 7 days. Cells upon passage 42 were applied in this study.

Immunocytochemistry

The expression of OCT2 and zonula occludens-1 (ZO-1) in ciPTEC monolayers on hollow fibers were investigated using the immunocytochemistry methodology as previously described by Jansen *et al.* [31]. The ZO-1 (Invitrogen, Carlsbad, CA) and OCT2 primary antibodies were diluted (1:50) in block solution (HBSS buffer supplemented with 2% (w/v) BSA, 2% (v/v) fetal calf serum and 0.001% (v/v) Tween-20 to prevent aspecific binding), followed by incubation with goat-anti-mouse-Alexa488 conjugate or goat-anti-rabbit-Alexa568 conjugate (1:200, Life Technologies), respectively. The expression of the ECM component collagen IV was investigated using the primary anti-type IV collagen antibody (1:50, Southern Biotech (Birmingham, AL)) followed by incubation with donkey-anti-goat-Alexa 488 conjugate ((1:200, Life Technologies).

Transmission- and scanning electron microscopy

The morphology of ciPTEC and its organelles was investigated using transmission electron microscopy (TEM) and SEM. In short for TEM, cells were primary fixed using 2% (w/v) glutaraldehyde, postfixated in 1% (w/v) osmiumtetroxide supplemented with 1% (w/v) potassium ferrocyanide, all dissolved in 0.1 M sodium cacodylatebuffer at room temperature (rT) for 1 hour. Next, samples were dehydrated through a graded series of ethanol and finally embedded in Epon and polymerized at 60°C. After ultrathin sectioning and contrasting with uranylacetate and leadcitrate, sections were analyzed using a transmission electron microscope JEOL JEM 1010 (Jeol, Akishima Tokyo, Japan) at an accelerating voltage of 60 kV and images were captured using a Kodak Megaplus camera (San Diego, CA). For SEM purposes, cells were washed twice in 0.1M sodiumcacodylate buffer supplemented with 3% (w/v) sucrose (SCS) and primary fixed using 2% (w/v) glutaraldehyde in SCS at rT for 1 hour. Next, cells were washed three times in SCS and secondly fixed in 1% (w/v) osmiumtetroxide in SCS at rT for 2 hours. After this, cells were incubated using 1% (w/v) tannic acid in SCS at rT for 1 hour. Subsequently, samples were washed three times, processed to 70% (v/v) ethanol and incubated in 5% (w/v) uranyl acetate dissolved in 70% (v/v) EtOH at rT overnight. Samples were dehydrated trough a graded series of ethanol (70%-80%-96%-100% (v/v)) and critical point dried. Finally, samples were gold sputtered prior to SEM analysis (JEOL JSM-6340F, Tokyo, Japan).

Transepithelial barrier function

Paracellular permeability was determined in the living membrane by endpoint quantification of inulin-fluorescein isothiocyanate (FITC) (0.1 $mg.ml^{-1}$ in Krebs-Henseleit buffer (containing $MgSO_4$ (0.14 g/L), KCl (0.35 g/L), KH_2PO_4 (0.16 g/L), NaCl (6.9 g/L), $CaCl_2$ (0.37 g/L), $NaHCO_3$ (2.1 g/L) and D-Glucose (2.0 g/L))

supplemented with HEPES (10 mM; KHH buffer)) diffusion when perfused (6 $ml.h^{-1}$) using a syringe pump (Terumo STC-521, Terumo Europe N.V., Leuven, Belgium) for 13 min at 37°C. The inulin-FITC diffusion in coated HFM in the absence of cells was investigated in similar conditions. During the experiment, the HFM was connected to a separated in- and outlet glass cannula (inner diameter 120 – 150 µm; DMT Trading, Aarhus, Denmark) assembled in a custom-made 3D printed cytocompatible polyester device (figure S1) to enable a separated basolateral (inner HFM, perfusion channel) and apical compartment (outer HFM, containing 300 µl KHH buffer). The inlet cannula was connected to a tubing system and a syringe pump, whereas the outlet cannula was connected to a tubing system and a depot to collect perfusate. First, the HFM was rinsed once using KHH buffer. Next, the inulin-FITC was perfused and 100 µl sample was collected in the apical compartment after 13 min.

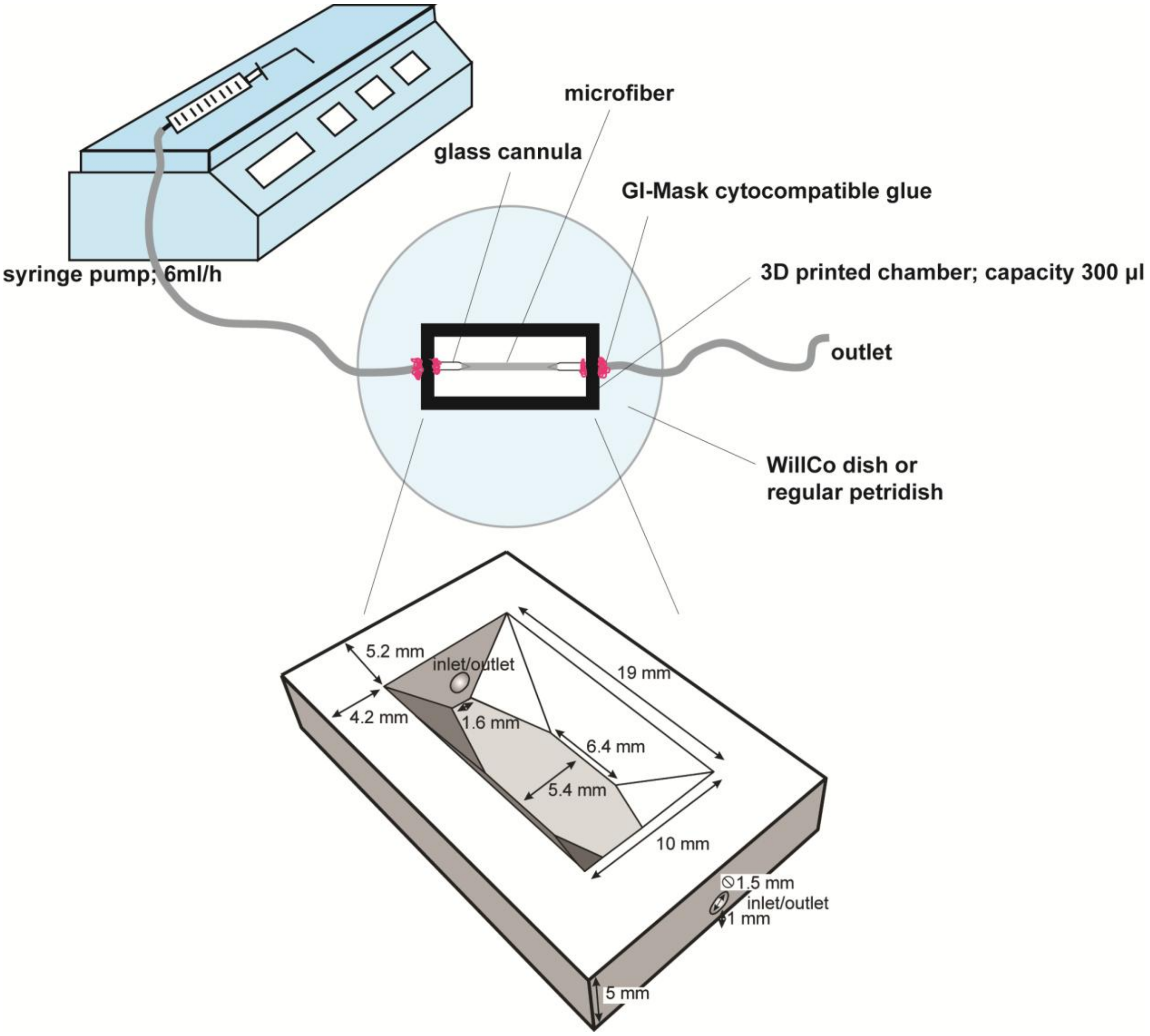

Supplemental figure S1. Schematic presentation of a flow experiment setup including a 3D-printed microfluidic chamber. The microfiber was connected to a separated in- and outlet glass cannula assembled in a custom-made 3D printed cytocompatible polyester device. The chamber was printed using a calibrated ROBO 3D beta R1 printer (Robo3D LLC, San Diago, CA) and the cytocompatible acrylonitril butadiene styrene (ABS) polymer. The ABS print temperature was 215°C on a heated bed of 90°C and the thickness of each layer was 100 µm, using a nozzle diameter of 0.3 mm. Dimensions as shown in the figure were optimized to have a stable system for fiber assembly and a low inner volume of 300 µl buffer, which enabled detection of inulin-FITC. Obviously, the buffer covered the fiber completely. For imaging purposes, the chamber was connected to a WillCo dish (WillCo Wells B.V., Amsterdam, The Netherlands) with a glass bottom using GI-MASK cytocompatible glue (Dental Union, Nieuwegein, The Netherlands) to prevent leakage from the chamber. During the experiment the system was closed using a cabinet in which 5% (v/v) CO_2 was present and the temperature was controlled at 37°C. The real-time imaging was performed using a 40x magnification water lens of the confocal microscope. To test paracellular permeability using inulin-FITC, the chamber was connected to a regular petridish and the temperature was controlled at 37°C during the experiment using a heating plate. In both types of experiments a flow rate of 6 $ml.h^{-1}$ was used for 13 min, using a syringe pump.

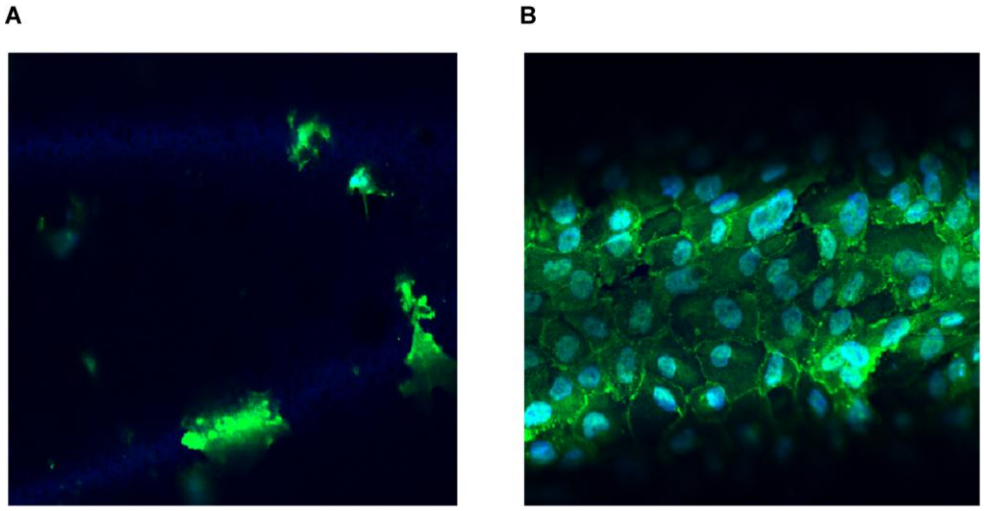

Supplemental figure S2. Representative images of ciPTEC performance on HCO 1100 and MicroPES HFM. (a) A poor cell adhesion was observed (i.e. no nuclei (blue)) when ciPTEC were cultured on double coated PES HCO 1100 HFM (inner diameter 215 µm). (b) Using a similar double coating procedure and the same batch of cells as used for HCO 1100 HFM, optimal cell adhesion and a tight monolayer formation with abundant ZO-1 expression (green) was detected using MicroPES HFM (inner diameter 300 µm).
